# The Financial Strength of Our Voluntary Charities

**Published:** 1895-01-12

**Authors:** B. Burford Rawlings


### The Institutional Workshop.

### THE FINANCIAL STRENGTH OF OUR VOLUNTARY CHARITIES.

By Mr. B. Burford Rawlings.

[This letter appeared in the *Times* on 9th inst.]

I have been hoping that some more powerful pen than mine would be exercised in reference to the two letters from Mr. Henry C. Burdett which recently appeared in your columns. In default of this, and lest any who read and were convinced by the first letter should have failed to notice the second, may I be permitted to recapitulate the circumstances?

On Christmas Day you, sir, published an article setting forth the urgent needs of the hospitals in language as timely as it was temperate. Two days after Mr. Burdett demurred to your conclusions and intimated that the hospitals are not in difficulties. In support of his contention he gave statistics exhibiting an increase in hospital work during the last decade of 14½ per cent., and an increase in their incomes during the same period of 40 per cent. How then could they be in difficulty? Where was the crisis of which the *Times* had written? That many of your readers would be reassured by this statement made by one who is, not unreasonably, regarded as an authority, and would in consequence withhold their contributions or divert them into other channels may be taken for granted.

On the 3rd inst., a full week afterwards, and a most important week to impecunious institutions, a second letter appeared from Mr. Burdett, wherein he expressed his regret "that in some way, for which I am unable to account, the income and expenditure figures have been reversed in the columns of the table I sent you."

To this frank avowal of error, which, though it could not, unhappily, repair the mischief wrought, would have disarmed criticism, Mr. Burdett adds the following remarkable statement: "It is satisfactory to find that the reversal of the figures in the table at first published does not upset my contention, for it merely diminishes the volume of the increase in the income of the hospitals, whilst the increase itself stands."

Mr. Burdett appears unwilling to let the hospitals have the benefit of his tardy correction. Can he seriously mean that his argument remains where it was now that the false foundation upon which he had unwittingly built it has disappeared—that there is no real difference between having received 30s. and spent 10s., as he first put it, and having received 10s. and spent 30s. as he now admits is the case?

It would be absurd to question Mr. Burdett's comprehensive grasp of his subject, yet it may be permitted to us to inquire whether he is not getting a little out of touch with the actual daily work of the hospitals and a little more dependent upon statistics; whether, as the past recedes, he is not learning to forget the struggles in which he once shared; and whether the humblest drudge of a secretary, who in somewhat impaired faith and with despairing zeal issues the monotonous appeal of which Mr. Burdett writes so scornfully, has not more real knowledge and is capable of more reliable testimony than one now far removed from the arena, even though a Past Grand Master of the Craft. I think, sir, that if the treasurers and secretaries of the metropolitan hospitals were referred to—although, of course, I have not the slightest mandate to write on any one's behalf but my own—they, with perhaps here and there an exception, could furnish information of almost unprecedented financial difficulty, such as would more than justify your sympathetic article.

If I had any doubt upon this point I should fall back upon the utterances of Mr. Burdett himself. I have a recollection of his vigorous speech to a meeting of experts called together by him some three years ago to devise means for assisting the finances of the metropolitan hospitals, when he drew a vivid picture of the straits they were in, and opined that in the absence of a more generous voluntary support an ultimate resort to Government or parochial assistance might become a real danger. The powerful book written by Mr. Egmont Hake under the inspiration of the committee then formed, and over which Mr. Burdett presided, had no other object than the awakening of the public to the sore needs of the hospitals and the danger of neglecting them, and in proportion to our sense of obligation to Mr. Burdett for the part he took then, involving as it did not only labour, but considerable expense, we must be surprised at his attitude now.

Has anything happened of late to make the hospital position financially better? Is it not the fact that it is worse? The Lords' Committee, while giving the hospitals high commendation, indicated many ways of spending money without showing them one way of getting it, and the result is reflected in Mr. Burdett's corrected statistics. But I question whether the figures, accurate though in a sense they may be, adequately represent the case for the hospitals, among other reasons because they may include windfalls to individual institutions, which in no way help the remainder. The weakness of hospital finance lies in the fact that nearly every institution must depend so largely upon a few generous friends to make up for a general neglect.

The question at the root of the matter is how the increase in the receipts, insufficient though it be, indicates an increased number of givers. Within my experience, which in length corresponds with that of Mr. Burdett, I have observed great changes in the volume and apportionment of gifts. Donations as a source of reliable income have almost wholly failed during recent years—I write now of the hospital I am privileged to serve—they are less in actual amount than when our expenditure was scarcely a fifth of what it is, and more than one-half the total has been contributed within any of the last six years by less than a dozen donors. Thus we become more and more dependent upon legacies and large benefactions to help meet the daily outgoings. That is to say, we have too often to go into debt, and are being taught to look to one donor or one testator to supply the deficiencies of 500, or it may be 5,000, members of the community, upon some of whom the hospital may have a far more direct claim than upon the donor of large amounts who has never made use of it.

Mr. Burdett deplores that hospital financiers "go over and over the same ground." Can he show us how to bring any other into cultivation? Three years ago he and others tried and failed. Charity authorities are always trying and failing. I have known an issue of 15,000 letters of appeal addressed, with pamphlets, to names taken from the Court Directory, to produce less than half the cost of printing and postage; while, as an extreme example on the other side, I have known an appeal with a personal element in it addressed to persons already givers, which has produced £2,000 at a cost of 20s. When hospitals advertise in the daily and weekly newspapers, it cannot be said they appeal exclusively to previous donors. Neither is the purview of their good work limited to their friends. Yet, who, besides previous donors to one charity or another ever responds, and what, it would be interesting to know, has been the general result of advertising during the past three weeks—a period at one time prolific? In our case, while we have spent £25 in advertising, and have admitted to in-treatment some 60 patients from all parts of the kingdom, we have received but £13 11s. towards a difference between assured income and expenditure during the same time amounting to £500.

If I have more respect for Mr. Burdett's influence than to suppose that the publication of his mistaken figures had no effect, I do not suggest that they are wholly responsible for the untoward result in our case, because the tendency has been in the same direction for years past. All must agree with him that the charity field is too restricted. Calculated by the rood or acre, the yield is generous beyond measure, but the area is insufficient; and whoever will show a way of enlarging it will be a welcome benefactor to all institutions "dependent upon voluntary contributions."

